# RIPS (rapid intuitive pathogen surveillance): a tool for surveillance of genome sequence data from foodborne bacterial pathogens

**DOI:** 10.3389/fbinf.2024.1415078

**Published:** 2024-08-09

**Authors:** Tim Muruvanda, Hugh Rand, James Pettengill, Arthur Pightling

**Affiliations:** Center for Food Safety and Applied Nutrition, Food and Drug Administration, College Park, MD, United States

**Keywords:** whole-genome sequencing, *Listeria* monocytogenes, *Salmonella enterica*, *Escherichia coli*, epidemiologic surveillance, outbreak detection, foodborne pathogens, food safety

## Abstract

Monitoring data submitted to the National Center for Biotechnology Information’s Pathogen Detection whole-genome sequence database, which includes the foodborne bacterial pathogens *Listeria* monocytogenes, *Salmonella enterica*, and *Escherichia coli*, has proven effective for detecting emerging outbreaks. As part of the submission process, new sequence data are typed using a whole-genome multi-locus sequence typing scheme and clustered with sequences already in the database. Publicly available text files contain the results of these analyses. However, contextualizing and interpreting this information is complex. We present the Rapid Intuitive Pathogen Surveillance (RIPS) tool, which shows the results of the NCBI Rapid Reports, along with appropriate metadata, in a graphical, interactive dashboard. RIPS makes the information in the Rapid Reports useful for real-time surveillance of genome sequence databases.

## Background

Whole-genome sequencing (WGS) helps investigators detect and investigate illness outbreaks caused by foodborne bacterial pathogens (Allard et al., 2016; Jackson et al., 2016; Pightling et al., 2018; Stevens et al., 2022; McClure et al., 2023). The National Center for Biotechnology Information (NCBI) provides a common WGS database (Pathogen Detection [PD] https://www.ncbi.nlm.nih.gov/pathogens/) (Sayers et al., 2023), to which federal, state, academic, and other laboratories from around the world may submit data (Stevens et al., 2017; Timme et al., 2019; Timme et al., 2020).

Analysts at the Food and Drug Administration (FDA) monitor the NCBI PD database to detect outbreaks and track trends (e.g., the emergence of strains of interest), evidence that is used for making decisions and improving emergency responses (US Food and Drug Administration, 2021; US Food and Drug Administration, 2022; Wellman et al., 2023). Since much of the data are submitted soon after collection, the database provides a near real-time view of pathogen distribution and genetic similarity. The NCBI’s *Listeria* monocytogenes, *S. enterica*, and *E. coli* data submission workflows employ a whole-genome multi-locus sequence typing scheme (wgMLST) to type sequences and identify closely related sequences already in the database (National Center for Biotechnology Information, 2023e). Text files called Rapid Reports provide the results of these preliminary analyses that are currently available only for L. monocytogenes, *Salmonella enterica*, and *Escherichia coli* (National Center for Biotechnology Information, 2023a; National Center for Biotechnology Information, 2023b; National Center for Biotechnology Information, 2023c). However, filtering the information contained in the reports and extracting the corresponding metadata that make it possible to search for actionable signals may be prohibitively complex for many.

We present RIPS (Rapid Intuitive Pathogen Surveillance), a user-friendly, accessible tool for retrieving Rapid Reports and associated metadata and filtering the data with user-prescribed conditions. With its interactive dashboard, RIPS is effective for monitoring the WGS database. Users can also utilize RIPS to examine sequences submitted on a given day and to determine what genome sequences in the database are closely related to data submitted by the user.

## Methods

RIPS is an open-source application written with the R Shiny package (Chang W et al., 2023). To install and run RIPS, users should use RStudio and the software available on GitHub (github.com/CFSAN-Biostatistics/RIPS). The install. R script will download and install all the dependencies required. To launch RIPS, open the app. R file and select the green “Run” on the top left of the script window. Further help can be found on the GitHub page.

### General processing workflow

**FIGURE 1:**
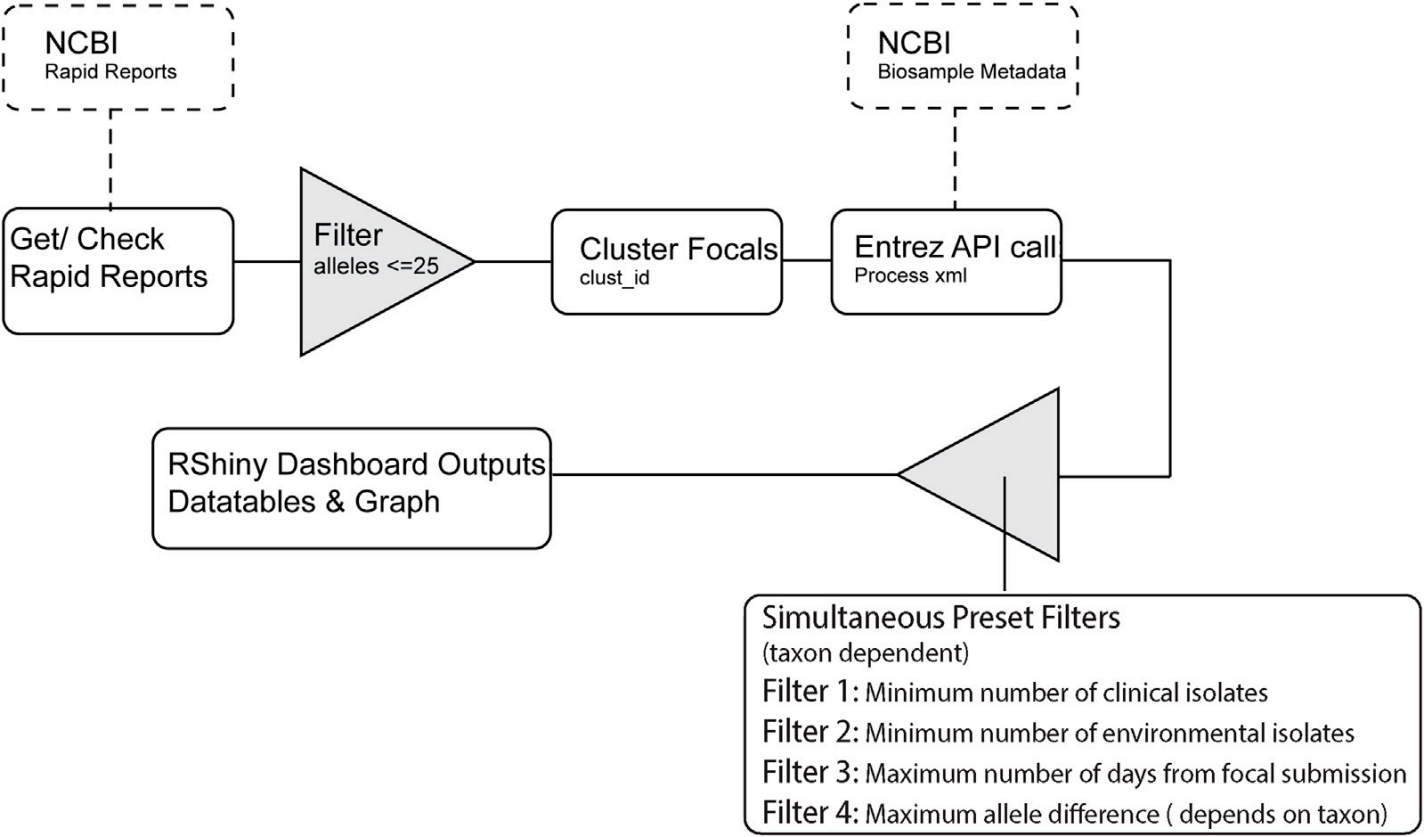
RIPS processing flowchart.

The full RIPS workflow for processing Rapid Reports can been seen in Figure 1. RIPS first downloads Rapid Reports and associated metadata files generated by the NCBI (see example Rapid Report in Supplemental Table S1). Users can filter the information using metrics, such as the number of sequences for bacteria collected from patients (clinical isolates) or food or environmental samples (non-clinical isolates) within specified timeframes and allele distances. RIPS displays the result on an interactive dashboard.

### Usage

Users should press the “Run/Get reports for new date” button to start the dashboard (Figure 2.1). Once fully loaded, an interactive bar graph is displayed showing the NCBI cluster and the number of isolates listed in the Rapid Report of that day (Figure 2.2). When a cluster on the bar graph is clicked, detailed metadata on the isolate of interest (focal isolate) is displayed, along with isolates closely related as determined by wgMLST allele difference (neighbor isolates; Figure 2.3).

Users can set the minimum criteria for clusters to be displayed on the bar graph (Figure 2.4). Default values of these filters follow Centers for Disease Control and Prevention recommendations (Besser et al., 2019; Leeper et al., 2023). For example, in Figure 2.4, the filter is set to show clusters that include a minimum of seven clinical isolates whose collection dates are within the last 60 days and have maximum genomic distances of ten alleles. We also added the condition that there be at least one environmental/other isolate.

### Rapid Reports and clustering

Each Rapid Report provides data for one genome sequence on the submission date and a list of “…the top 5 nearest neighbors, and all neighbors <6 allele differences away” with allele distances, BioSample numbers, isolate identifiers, and cluster accession numbers (National Center for Biotechnology Information, 2023e).

Rapid Reports for each submitted sequence are provided in separate reports, even if they are identical to other sequences or are part of a batch of submitted sequences. Therefore, to identify multiple recently submitted sequences closely related to each other and their neighbors, RIPS clusters focal isolate sequences together if they share one or more neighboring sequences or if the submitted sequences appear as neighbors to each other. For example, given the Rapid Report for two genomes, A and B, that fall into the same NCBI cluster, the Rapid Report for genome A, containing neighboring sequences X, Y, and Z, would not cluster with the Rapid Report for genome B containing neighboring sequences Q, R, and S. If, however, the Rapid Report for genome B included neighboring sequences Q, R, and X, then the two genomes A and B would be clustered together since they share the neighboring sequence X. This step groups newly submitted sequences that are closely related (as determined by the allele distance prescribed by the user) and separates newly submitted sequences into different groups if they occupy different parts of the same NCBI cluster.

### Metadata capture

RIPS uses an Application Programming Interface (API) call with NCBI’s Entrez Programming Utilities or E-utility to retrieve relevant metadata from the NCBI BioSample database. A unique NCBI developer API key is required for this functionality and is freely available at the NCBI through registration. By default, without an API key, the E-utility limits API requests to three per second. With an API key, this limit is ten per second (National Center for Biotechnology Information, 2017; Sayers, 2009). Users should select the prior day to ensure that metadata from the BioSample database will be available and complete. The age of each isolate is calculated based on the availability of the metadata, including the submission date, collection date, and creation date. Each Entrez API call results in an XML document containing the metadata that describes the BioSamples from the list, handled by the function “get_samn_info2.” If BioSample data are not available, RIPS will estimate the submission date using the Rapid Report date as the submission date.

### Dashboard features: Data filtration, bar graph and metadata tables

RIPS allows for filtering the Rapid Reports by 1) a minimum number of clinical isolates, 2) a minimum number of non-clinical isolates, 3) a maximum number of days from subject submission, and 4) maximum allele difference. RIPS displays clusters with an interactive bar graph. Clicking on bars displays metadata information of the sequences in the cluster. Visualizations for the bar graph are coded using the ggplot2 and ggplotly libraries (Wickham, 2016; Sievert, 2020).

Below the graph are two tables, the Focal Isolates table and Neighbor isolates table. The Focal Isolates table contains all the new isolates that triggered the Rapid Report. In the Neighbor Isolates table, all available metadata on isolates in NCBI are shown and can be viewed by scrolling right on the horizontal scroll bar or keyboard. Blue buttons in each table are external NCBI search links to BioSample entries or the Pathogen Detection browser. There are two tabs for displaying isolate information. The default ‘Main View’ tab contains the previously mentioned Focal and Neighbors table. The alternate ‘All data’ tab shows metadata for all isolates visible in the bar graph and includes a search box. The search box allows users to search any term across all fields in the table. Combining this search functionality with filtering enables users to search for terms and view recent matches.

**FIGURE 2:**
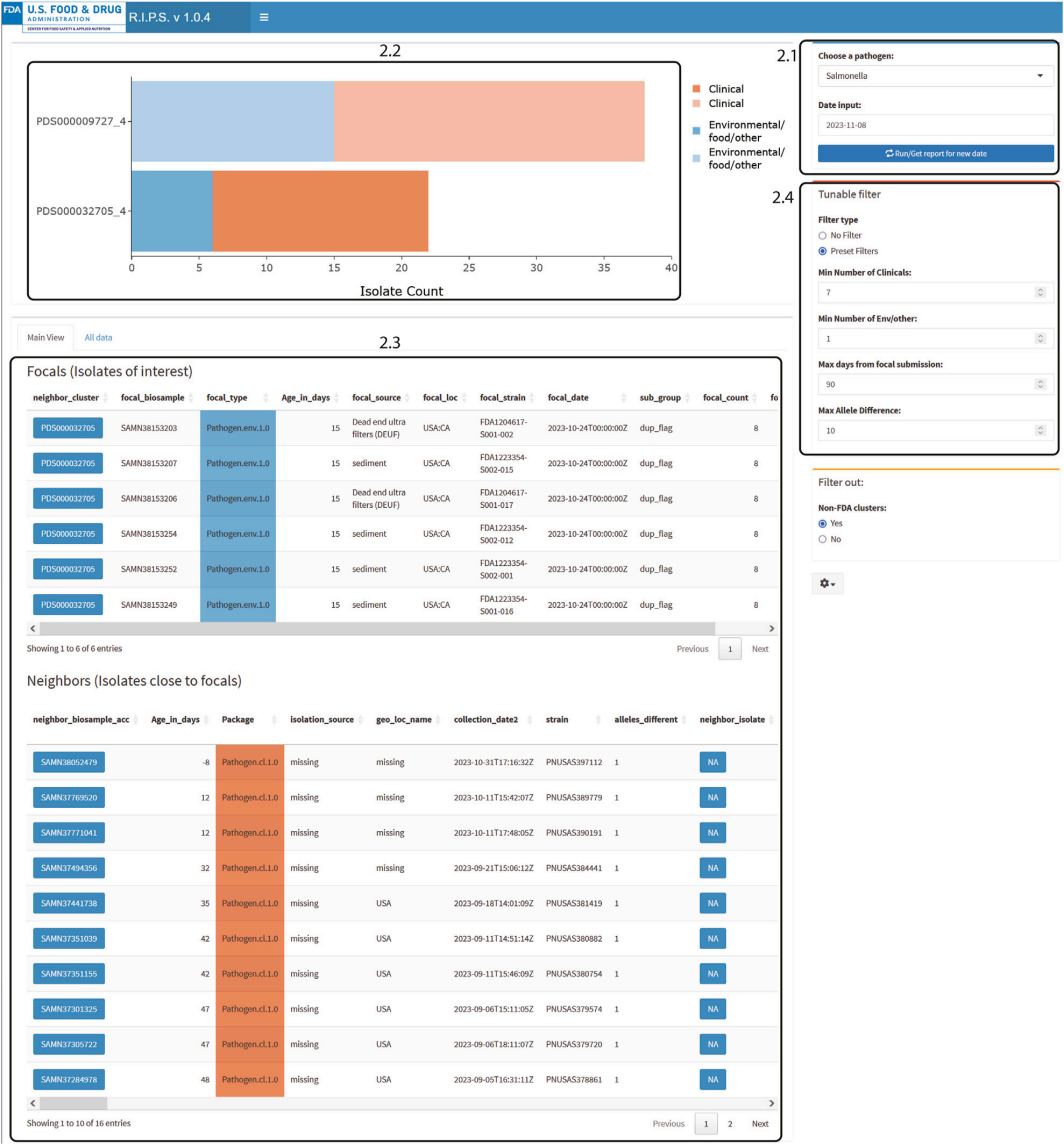
RIPS output for filtered query of *Salmonella* database on 8 November 2023. Filter was set for at least seven clinical isolates within 10 alleles submitted during the prior 60 days and at least one matching non-clinical isolate.

Users can download a CSV file containing detailed information on the highlighted cluster using the ‘Download Report’ button in the settings (Figure 2, gear icon).

## Results and discussion

The RIPS software is lightweight and not compute intensive. The utility of RIPS comes from integrating isolate metadata to Rapid Report data while incorporating filtering options. The raw Rapid Report data for a genome sequence are hard to interpret. For example, for the Rapid Report of genome sequence with NCBI accession number SRR26715329 (Supplemental Table S1), users can only determine how far apart a neighboring isolate is to the focal isolate and the NCBI cluster in which it is located. Comparatively, for the same Rapid Report (Supplemental Table S1), when processed through RIPS, further context has been added (Figure 2). Here, RIPS has grouped five other similar sequences with SRR26715329 (identifier FDA1204617-S001-002) in the NCBI cluster by gathering all the focal isolates that contain similar neighbors. Additionally, metadata fields such as isolation source and collection date allow users to view this information and quickly make decisions on whether clusters warrant further investigation. For example, (Figure 2), the metadata for the six FDA isolates shown indicates that the isolates were obtained from sediment or dead-end ultra filtration samples and are one allele distant from at least ten clinical isolates seen in the Neighbors table (Besser et al., 2019). Since the clinical isolates are one allele distant from the environmental isolates, the source of the environmental isolates can also be a potential source for the clinical isolates.

**FIGURE 3:**
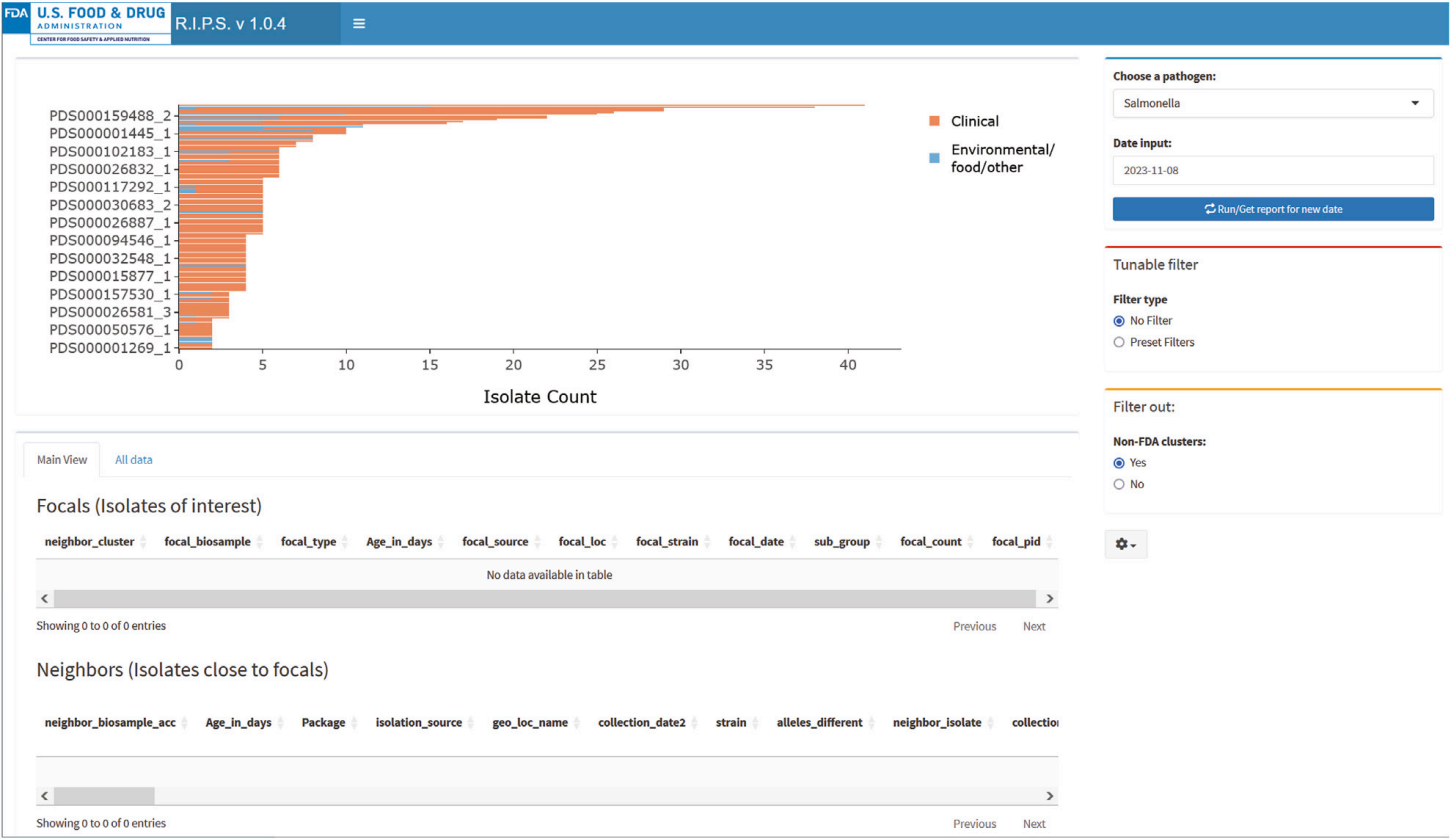
RIPS output for unfiltered query of *Salmonella* database on 8 November 2023.

Figures 2, 3 demonstrate the effectiveness of RIPS for monitoring the genome sequence database for emerging outbreaks. We analyzed Rapid Reports for sequences that the FDA submitted during a foodborne illness outbreak investigation (US Food and Drug Administration, 2023). In November 2023, the FDA investigated an outbreak of illnesses caused by onions contaminated with *Salmonella*. As part of the investigation, FDA inspectors collected samples from a farm that tested positive for *Salmonella* Thompson. Lab technicians submitted genome sequence data to the NCBI PD database on 8 November 2023. The unfiltered results show many genome sequence submissions on that date (Figure 3). With filtering, RIPS removes all but two clusters (Figure 2). The user can further investigate the clusters by clicking on the bars and generating a list of the genomes in the cluster with associated metadata. While both filtered clusters include sequences submitted as part of the outbreak investigation, the cluster labeled PDS000032705_4 includes six FDA genome sequences that match the genomes of 16 clinical isolates that are part of the outbreak (Pightling et al., 2018).

**FIGURE 4:**
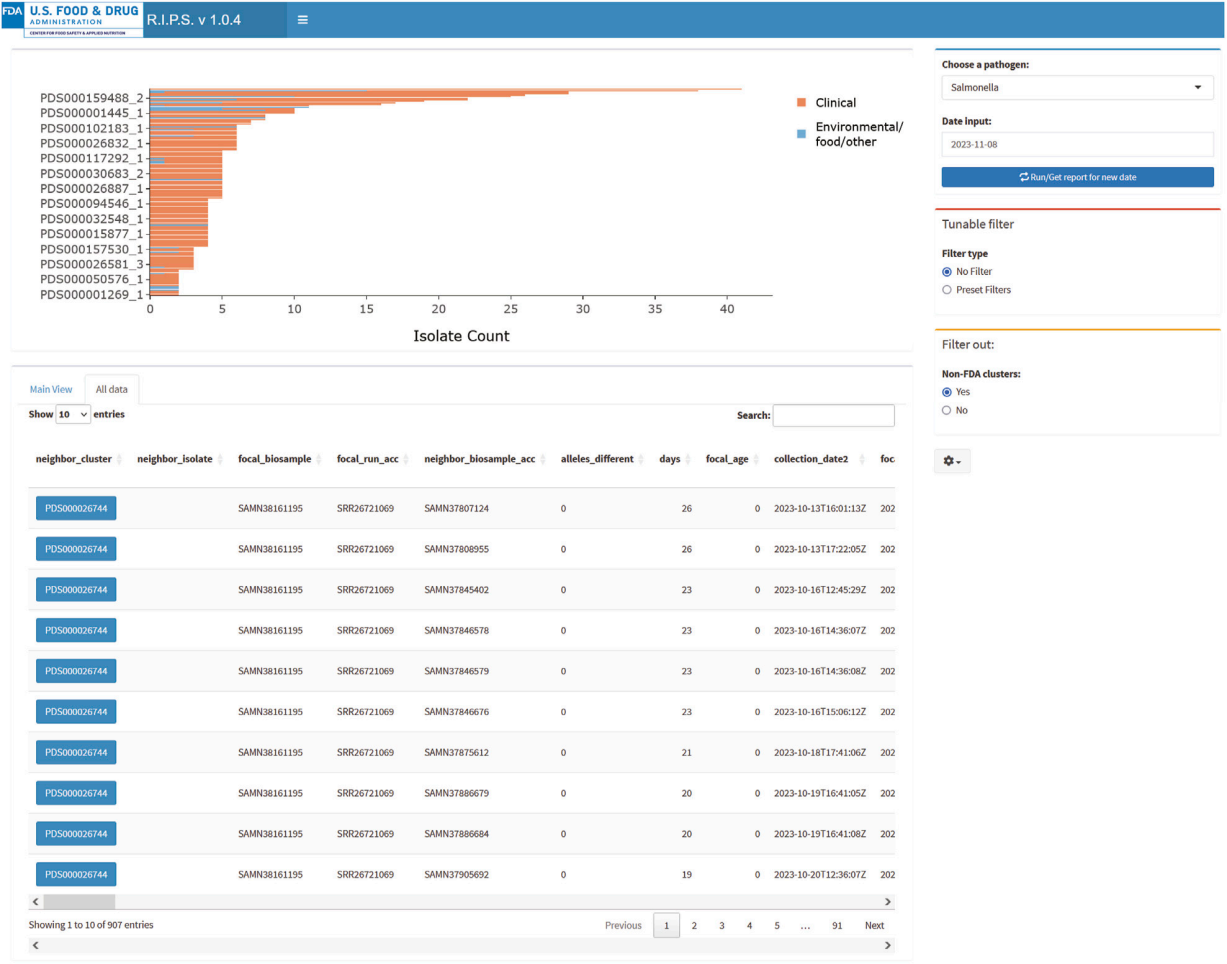
RIPS output for query of *Salmonella* database on 8 November 2023, in “All data” view.

Another function of RIPS is to allow users to query the Rapid Reports using known sequence identifiers. The “All data” view presents the identities of all sequence data submitted, matching sequences contained within the Rapid Reports and the NCBI cluster identifiers. For example, during the above investigation, the FDA submitted sequence data with the identifier “FDA1204617-S001-002.” Entering the identifier in the search field retrieves metadata for sequences in the associated Rapid Report (Figure 4).

Rapid Reports provide immediate estimates of genomic distances intended to be used to assign newly submitted data to clusters, and the reports may not show all the data available in the database. Furthermore, wgMLST measurements are unfiltered and may differ from NCBI’s SNP analyses, which undergo SNP density filtering (National Center for Biotechnology Information, 2023e). For these reasons, Rapid Reports are best used as companions to the Pathogen Detection browser, which illustrates relationships between genomes with interactive trees (National Center for Biotechnology Information, 2023d).

## Conclusion

RIPS makes it easier to monitor foodborne bacterial pathogens with Rapid Reports by filtering the results of wgMLST analyses performed as part of the NCBI’s PD genome sequence submission workflow. RIPS allows for the use of uniform criteria that can be adjusted for optimal performance. By presenting the results provided in text files in an accessible graphical interface, RIPS provides a means of quickly detecting signals and initiating actions in response to information submitted to the whole-genome sequence database.

## Data Availability

The original contributions presented in the study are included in the article/Supplemental Material, further inquiries can be directed to the corresponding author.

## Abbreviations

NCBI, National Center for Biotechnology Information; wgMLST, whole-genome multi locus sequence typing; SNP, single nucleotide polymorphism; WGS, whole-genome sequencing; FDA, US Food and Drug Administration.
