# Supplementary material for: RIPS (rapid intuitive pathogen surveillance): a tool for surveillance of genome sequence data from foodborne bacterial pathogens
Source: Front Bioinform. 2024 Aug 9;4:1415078. doi: 10.3389/fbinf.2024.1415078 (PMC11341538; doi:10.3389/fbinf.2024.1415078)
Supplement: Supplementary file 1 [file Table1.pdf]

Supplemental Table 1: Rapid Report for genome sequence submitted by the FDA (NCBI accession number SRR26715329 [identifier FDA1204617-S001-002]).

#biosample_acc	run_acc	neighbor_biosample_acc	neighbor_run_acc	neighbor_assembly_acc	neighbor_isolate	neighbor_cluster	alleles_different	loci_in_common
SAMN38153203	SRR26715329	SAMN38123314	SRR26686007	NULL	PDT001972479	PDS000032705	0	4383
SAMN38153203	SRR26715329	SAMN38123312	SRR26686002	NULL	PDT001972477	PDS000032705	0	4382
SAMN38153203	SRR26715329	SAMN37771041	SRR26352875	NULL	PDT001924829	PDS000032705	1	4385
SAMN38153203	SRR26715329	SAMN38052479	SRR26621042	NULL	PDT001963553	PDS000032705	1	4385
SAMN38153203	SRR26715329	SAMN37154956	SRR25760329	NULL	PDT001862475	PDS000032705	1	4384
SAMN38153203	SRR26715329	SAMN37233282	SRR25874306	NULL	PDT001871506	PDS000032705	1	4384
SAMN38153203	SRR26715329	SAMN37769520	SRR26351571	NULL	PDT001924748	PDS000032705	1	4384
SAMN38153203	SRR26715329	SAMN37494356	SRR26135143	NULL	PDT001892922	PDS000032705	1	4384
SAMN38153203	SRR26715329	SAMN37284978	SRR25917057	NULL	PDT001873651	PDS000032705	1	4384
SAMN38153203	SRR26715329	SAMN37185538	SRR25779770	NULL	PDT001868309	PDS000032705	1	4383
SAMN38153203	SRR26715329	SAMN37351039	SRR26028065	NULL	PDT001881336	PDS000032705	1	4383
SAMN38153203	SRR26715329	SAMN37216956	SRR25837838	NULL	PDT001870107	PDS000032705	1	4383
SAMN38153203	SRR26715329	SAMN37185529	SRR25779782	NULL	PDT001868321	PDS000032705	1	4383
SAMN38153203	SRR26715329	SAMN37233288	SRR25872630	NULL	PDT001871182	PDS000032705	1	4382
SAMN38153203	SRR26715329	SAMN37301325	SRR25929988	NULL	PDT001876016	PDS000032705	1	4381
SAMN38153203	SRR26715329	SAMN37232851	SRR25871358	NULL	PDT001870849	PDS000032705	1	4381
SAMN38153203	SRR26715329	SAMN37305722	SRR25932394	NULL	PDT001876370	PDS000032705	1	4380
SAMN38153203	SRR26715329	SAMN37232852	SRR25871357	NULL	PDT001870848	PDS000032705	1	4379
SAMN38153203	SRR26715329	SAMN37351155	SRR26030639	NULL	PDT001881512	PDS000032705	1	4378
SAMN38153203	SRR26715329	SAMN37494356	NULL	GCA_031896195.1	PDT001892922	PDS000032705	1	4350
SAMN38153203	SRR26715329	SAMN37305722	NULL	GCA_031205165.1	PDT001876370	PDS000032705	1	4315
SAMN38153203	SRR26715329	SAMN37301325	NULL	GCA_031217635.1	PDT001876016	PDS000032705	1	4309
SAMN38153203	SRR26715329	SAMN37351039	NULL	GCA_031355215.1	PDT001881336	PDS000032705	1	4266
